# Evaluating Effect Moderators in Cognitive Versus Behavioral Based CBT-Modules and Sequences Towards Preventing Adolescent Depression

**DOI:** 10.1080/15374416.2023.2209181

**Published:** 2023-05-22

**Authors:** Marieke W. H. van den Heuvel, Denise H. M. Bodden, Filip Smit, Yvonne A. J. Stikkelbroek, Rutger C. M. E. Engels

**Affiliations:** a Erasmus School of Social and Behavioural Sciences, Erasmus University Rotterdam; b Department of Mental Health and Prevention, Trimbos-Institute, Netherlands Institute of Mental Health and Addiction; c Department of Child and Adolescent Studies, Utrecht University; d Department of Clinical Psychology and Department of Epidemiology and Biostatistics, Amsterdam University Medical Centers, Location VUmc; e Depression Expert Center for Youth, Mental Health Care Oost-Brabant

## Abstract

**Objective:**

The aim of this study was to investigate age group, gender, and baseline depressive symptom severity as possible effect moderators in (1) cognitive versus behavioral based CBT-modules and (2) sequences of modules that started either with cognitive or behavioral modules in indicated depression prevention in adolescents.

**Method:**

We conducted a pragmatic cluster-randomized trial under four parallel conditions. Each condition consisted of four CBT-modules of three sessions (cognitive restructuring, problem solving, behavioral activation, relaxation), but the sequencing of modules differed. The CBT-modules and sequences were clustered into more cognitive versus more behavioral based approaches. The sample involved 282 Dutch adolescents with elevated depressive symptoms (Mage = 13.8; 55.7% girls, 92.9% Dutch). Assessments were conducted at baseline, after three sessions, at post-intervention and 6-month follow-up with self-reported depressive symptoms as the primary outcome.

**Results:**

We found no evidence for substantial moderation effects. Age group, gender, and depressive symptom severity level at baseline did not moderate the effects of cognitive versus behavioral modules after three sessions. No evidence was also found that these characteristics moderated the effectiveness of sequences of modules that started either with cognitive or behavioral modules at post-intervention and 6-month follow-up.

**Conclusion:**

Cognitive and behavioral based modules and sequences in the prevention of depression in adolescents might apply to a relatively wide range of adolescents in terms of age group, gender, and severity levels of depressive symptoms.

**Abbreviation:** CDI-2:F: Children’s Depression Inventory-2 Full-length version; CDI-2:S: Children’s Depression Inventory-2 Short version; STARr: Solve, Think, Act, Relax, and repeat

Cognitive Behavioral Therapy (CBT) is a commonly used approach to prevent depression in adolescents and has generally been shown to be effective in reducing depressive symptoms and decreasing the risk of developing a depressive disorder (e.g., Horowitz & Garber, 2006; Oud et al., 2019). Specifically, indicated prevention appears to be effective, but effect sizes are modest, and often fade over time (Cuijpers et al., 2021; Eckshtain et al., 2020; Rasing et al., 2017; Ssegonja et al., 2019). In addition, reviews have shown heterogeneity in outcomes, with some participants benefiting more than others (e.g., Conejo-Cerón et al., 2020; Stice et al., 2009).

CBT-based prevention programs targeting depression usually consist of a combination of components (e.g., McCarty & Weisz, 2007; Weersing et al., 2009). Based on cognitive and behavioral theories that are the basis of CBT (Beck et al., 1979; Lewinsohn, 1974), these components can be classified into two types, namely components consisting of mainly cognitive techniques (e.g., changing thoughts, beliefs or ways of thinking about the self, the world, the future, or situations/problems) and components consisting of mainly behavioral techniques (e.g., scheduling pleasant activities and exercises that promote relaxation) (e.g., Hetrick et al., 2014; Weersing et al., 2009).

Elsewhere, we examined the differential effectiveness of four CBT-components (operationalized as modules of three sessions), with two modules based mainly on the cognitive approach, namely Think (cognitive restructuring) and Solve (problem solving), and two modules based mainly on the behavioral approach, namely Act (behavioral activation) and Relax (relaxation) (Van den Heuvel et al., 2021). The differential effectiveness of four different sequences of these modules (Think-Act-Relax-Solve, Act-Think-Relax-Solve, Solve-Act-Think-Relax, and Relax-Solve-Act-Think) was also examined (Van den Heuvel et al., 2021). We found neither significant differences in effect between the four modules (after three sessions) nor between the four sequences at post-intervention and 6-month follow-up. However, these results might not apply to all adolescents – some might benefit less or more from specific modules and sequences than others.

The aim of the current study was to investigate the effect moderators of separate CBT-modules, classified into modules with a more cognitive orientation (Think and Solve) versus modules with a more behavioral orientation (Act and Relax). This classification was based on the intended change by the modules (i.e., expected mediating mechanisms). We also examined moderators in the effects of sequences starting with a cognitive module (*Think*-Act-Relax-Solve and *Solve*-Act-Think-Relax) versus sequences starting with a behavioral module (*Act*-Think-Relax-Solve and *Relax*-Solve-Act-Think). We examined age group (11–14 versus 15–18 years of age), gender, and initial severity of depressive symptoms as potential moderators. To date, no research has been conducted on the moderating role of these variables in the effect of individual CBT components and different sequences in adolescent depression prevention, but there are some studies on moderators in the effect of CBT programs as a whole package.

For example, various RCTs indicate that *age* is not a moderator in the effect of (group) CBT-based indicated prevention programs aimed at adolescent depression when compared to CBT-based bibliotherapy, a group support program, or an educational brochure (Brière et al., 2014; Conejo-Cerón et al., 2020; Müller et al., 2015; Stice et al., 2010). Also *gender* does not seem to moderate the effect of group CBT compared to these control conditions (Brière et al., 2014; Müller et al., 2015; Stice et al., 2010) and an individual support program (Duong et al., 2016). With one exception, among a sample of early adolescents (11–12 years) with elevated depressive symptoms, the Penn Resilience Program (group CBT) was more effective for girls than for boys relative to care as usual (Gillham et al., 2006). Evidence on the moderating role of *depressive symptom severity* on the effect of CBT is inconsistent (Conejo-Cerón et al., 2020). Some prevention studies show no moderating role of initial severity on the effect of CBT when compared to controls (e.g., CAU, individual support, and educational brochure) (Duong et al., 2016; Garber et al., 2009; Gau et al., 2012; Gillham et al., 2006), while other studies do, however in opposite directions (e.g., Müller et al., 2015; Weersing et al., 2016). Müller et al. (2015) revealed that, at post-intervention, the effects of indicated group CBT and CBT-based bibliotherapy were larger for adolescents with at least moderate levels of depressive symptoms compared to an educational brochure but not at follow-up. Weersing et al. (2016) found that the effect of CBT was smaller among adolescents with higher levels of depressive symptoms compared to CAU (in a sample of adolescents at risk for depression because of parental history of depression and/or having subclinical levels of depressive symptoms themselves).

Thus, to date, no (clear) moderation effects have been identified for CBT programs compared to other interventions related to age, gender, and symptom severity. This might be due to the fact that some of these trials have insufficient power to detect moderation. Another explanation for these inconclusive results could lie in the large differences between CBT programs in terms of components, dosage, frequency, modality, et cetera. Rather than focusing on CBT in a broad sense (a “blubber” of components), the current study distinguished between cognitive versus behavioral modules, and a cognitive versus a behavioral onset, because we expected certain groups to benefit more from one approach than the other. We investigated three adolescents’ characteristics as potential moderators, namely age group, gender, and symptom severity. The results may provide insights into which subgroups of adolescents benefit more or to a lesser extent from predominantly cognitive versus predominantly behavioral approaches in adolescent depression prevention. This knowledge is relevant as it can be used to redesign (specific components of) the intervention for subgroups for whom the intervention is ineffective or even has significant iatrogenic effects and/or to refer only to subgroups being most responsive to (specific components of) the intervention (Kraemer et al., 2002).

No research has been conducted on moderators in the effect of specific CBT components or sequences. Therefore, our hypotheses are mainly based on developmental theories. It could be expected that younger adolescents benefit less from cognitive approaches, because in early adolescence the cognitive (e.g., metacognition, abstract reasoning, and executive functioning skills such as planning and problem solving), social (e.g., perspective taking), and emotional skills (e.g., emotion understanding) that are necessary to understand and apply abstract concepts inherent to cognitive techniques have not been fully developed yet (Garber et al., 2016; Hetrick et al., 2014). Therefore, we hypothesized that compared to older adolescents (aged 15–18), *young adolescents* (aged 11–14) will benefit more from *modules with a behavioral approach* than from modules with a cognitive approach and more from *sequences of modules starting with a behavioral approach* than from sequences of modules starting with a cognitive approach.

Further, studies have shown gender differences in the expression of depression in adolescents, with girls reporting more cognitive and somatic symptoms of depression (e.g., excessive guilt, concentration difficulties, body image dissatisfaction, and eating problems) and boys reporting more behavioral symptoms (e.g., anhedonia, irritability, and morning fatigue) (Bennett et al., 2005). Based on these differences, we hypothesized that compared to boys, *girls* will benefit more from *modules with a cognitive approach* than from modules with a behavioral approach and from *sequences of modules starting with a cognitive approach* than from sequences of modules starting with a behavioral approach.

Finally, subclinical depression is a condition in which an individual has some depressive symptoms but does not meet the diagnostic criteria for a depressive disorder (Cuijpers & Smit, 2008). The severity of subclinical depression is commonly assessed with the aid of depression rating scales and cutoff scores, classifying depression symptoms into subclinical and clinical scores (Cuijpers & Smit, 2008; Klein, 2008). CBT treatment protocols for clinically depressed adolescents typically start with behavioral techniques (such as mood monitoring and pleasant activity scheduling) in order to improve current mood (Weersing et al., 2015). In addition, anhedonia (loss of pleasure in usual activities), a symptom that is more common and also more severe in adolescents with clinical levels of depressive symptoms than in adolescents with subclinical levels of depressive symptoms, is usually addressed through behavioral activation (Craske et al., 2016, 2019; Kennard et al., 2005). Therefore, we hypothesized that compared to adolescents with subclinical levels of symptoms, *adolescents with clinical levels of symptoms* will benefit more from *modules with a behavioral approach* (especially Act) than from modules with a cognitive approach and from *sequences of modules starting with a behavioral approach* (especially *Act*-Think-Relax-Solve) than from sequences starting with a cognitive approach.

## Methods

This study was approved by the medical ethics committee CMO Region Utrecht in The Netherlands (NL59152.041.16) and was registered in the Dutch Trial Register (Trial NL5584/NTR6176). It has been reported in accordance with the CONSORT 2010 statement for parallel group randomized trials (Schulz et al., 2010) and its extension to cluster randomized trials (Campbell et al., 2012).

### Trial Design

This study is part of a pragmatic non-masked multisite cluster-randomized prevention trial with four parallel conditions. See Van den Heuvel et al. (2019) for the study protocol and Van den Heuvel et al. (2021) for the main effects. We developed four CBT-modules of three sessions each, namely Think (cognitive restructuring), Act (behavioral activation), Solve (problem solving), and Relax (relaxation) and manipulated their sequencing. The conditions were as follows:

- Condition 1: Think-Act-Relax-Solve;
- Condition 2: Act-Think-Relax-Solve;
- Condition 3: Solve-Act-Think-Relax;
- Condition 4: Relax-Solve-Act-Think.

For a theoretical foundation of the four conditions, we refer to Van den Heuvel et al. (2021).

Assessments were conducted at baseline, after the first module, second and third modules (intermediate assessments 1, 2, and 3), after the fourth module (post-intervention) and at 6-month follow-up. In the current study, we used data of baseline, intermediate assessment 1, post-intervention, and 6-month follow-up. Participants received gift vouchers as reward for completing the assessments.

### Procedure

A total of 8,603 adolescents from 11 secondary schools in the Netherlands, from pre-vocational training up to pre-university level, were screened on depressive symptoms with the Child Depression Inventory-2 (CDI-2; Bodden et al., 2016; Kovacs, 2011) between 2017 and 2019. Inclusion criteria were ages between 10 and 20, sufficient knowledge of the Dutch language and elevated depressive symptoms according to the screening (percentile score >75 on CDI-2). Exclusion criteria were the absence of adolescents’ consent (or parental consent for adolescents aged younger than 16), currently in treatment for mood- or anxiety problems, or *acute* suicidal ideation (i.e., concrete plans). Participants who presented acute suicidal ideation (at any time point in the study) were referred to a GP within 48 h. All 2,009 adolescents (23.4%) who met the inclusion criteria received written information regarding the study, along with their parents if aged younger than 16. Subsequently, adolescents (and their parents) were contacted by the research team to ask for their consent and exclusion criteria were checked. Finally, 282 adolescents (14%) participated who all provided informed consent (and their parents for adolescents aged younger than 16). Adolescents were less likely to participate if they had a lower level of depressive symptoms (*OR* = 0.95, *p* < .001, 95% CI = 0.93–0.97), were older (*OR* = 1.17, *p* < .001, 95% CI = 1.08–1.27), had a low or moderate educational level (*OR* = 1.34, *p* = .028, 95% CI = 1.03–1.73), or had a non-Dutch ethnicity (*OR* = 2.76, *p* < .001, 95% CI = 1.71–4.46). Participants were stratified by gender (boys and girls) and age (11–13, 14–15, and 16–18 years) per school. Subsequently, treatment groups (52 in total) were formed of approximately five students (*M* = 5.42, *SD* = 1.41) from the same school, which were randomized as a cluster to one of the four conditions via computer-generated block randomization (block size four) by the first author. The four conditions consisted of, respectively, 14, 13, 13, and 12 clusters and 81, 69, 77, and 55 participants. Participants were informed of group allocation after the baseline measurement. More information about the procedure and participants’ flow is provided in Van den Heuvel et al. (2021).

### Participants

The participants were 282 adolescents (55.7% girls) with elevated levels of depressive symptoms, aged 11–18 years (*M* = 13.82, *SD* = 1.48). The majority of the participants were of Dutch ethnicity (92.9%). Educational levels were as follows: pre-vocational (6.7%), higher general (42.9%), or pre-university level (50.4%). Demographic variables per condition are reported in Table 1.

**Table 1.** Observed demographic variables and depression severity per approach (cognitive or behavioral) and per condition.

	Cognitive approaches		Behavioral approaches	
Variable	Condition 1: Think-Act-Relax-Solve (*n* = 81)	Condition 3: Solve-Act-Think-Relax (*n* = 77)	Condition 2: Act-Think-Relax-Solve (*n* = 69)	Condition 4: Relax-Solve-Act-Think (*n* = 55)
	*M (SD)*	*M (SD)*	*M (SD)*	*M (SD)*
Age (years)	13.88 (1.56)	13.95 (1.49)	13.62 (1.45)	13.78 (1.41)
	*n* (%)	*n* (%)	*n* (%)	*n* (%)
Gender				
Girls	43 (53.1)	40 (51.9)	40 (58.0)	34 (61.8)
Boys	38 (46.9)	37 (48.1)	29 (42.0)	21 (38.2)
Ethnicity				
Dutch	78 (96.3)	71 (92.2)	65 (94.2)	48 (87.3)
Other	3 (3.7)	6 (7.8)	4 (5.8)	7 (12.7)
Education level				
Low	0	1 (1.3)	14 (20.3)	4 (7.3)
Moderate	28 (34.6)	42 (54.5)	27 (39.1)	24 (43.6)
High	53 (65.4)	34 (44.2)	28 (40.6)	27 (49.1)
Depression severity				
CDI-2:F < 14	31 (38.3)	30 (39.0)	28 (40.6)	23 (41.8)
CDI-2:F ≥ 14	43 (53.1)	43 (55.8)	34 (49.3)	28 (50.9)
Missing	7 (8.6)	4 (5.2)	7 (10.1)	4 (7.3)

*Note*. Low: pre-vocational education (in Dutch: vmbo-basis/kader/gl), moderate: higher general secondary education (in Dutch: vmbo-tl, vmbo-tl/havo, havo), high: pre-university education (in Dutch: havo/vwo, vwo). CDI-2:F: Children’s Depression Inventory-2 Full-length version.

### Sample Size and Power

Originally, the study was powered to detect a main effect of *d* ≥ 0.33 (standardized mean difference of a size, which is clinically relevant cf. Lipsey & Wilson, 1993) at α ≤ 0.05 (2-tailed) with a power of (1-β) ≥ 0.80, while taking into account a design effect of 1.22 stemming from the participants being clustered in treatment groups (with a mean of 5 persons per group, a group size variation of 0.30 and an intraclass correlation of 0.05) and the correlation of the outcome (CDI-2) between baseline and intermediate assessment 1 of *r* = 0.80. This required *n* = 64 per trial arm (Van den Heuvel et al., 2019). We were mindful that the evaluation of moderation typically requires a fourfold sample size. Since the trial has four conditions, hence *N* = 256, we assumed that the trial would also be well-powered for moderation analysis.

The latter assumption was cross-checked in a post hoc power calculation for the moderated regression of the outcome Y (CDI-2) at follow-up t on condition C, moderator M, and their product CM, while holding constant for Y as measured at baseline: Y_t_ = a + b_1_C + b_2_M + b_3_CM + b_4_Y_t–1_, where C is the four conditions collapsed into two (behavior focused versus cognition focused interventions), and M is a binary moderator (e.g., boys versus girls). G*Power suggested that an interaction effect of size *f* = 0.23 (equivalent to *d* = 0.46 which is an effect of medium size) can be detected as statistically significant in a total sample size of *N* = 231. Multiplying by the design effect of 1.22, this becomes *N* = 282, which is exactly the sample size that was obtained.

### Interventions

The four modules were developed in collaboration with certified CBT-therapists and experts in the field and are based on CBT-theories and current CBT-protocols (e.g., The D(o)epression course (Stikkelbroek et al., 2005) and Modular Approach to Therapy for Children with Anxiety, Depression, or Conduct Problems (MATCH) (Chorpita & Weisz, 2009)). This resulted in the STARr-training, a CBT-based indicated prevention group program with four modules, namely Solve, Think, Act, and Relax (acronym of STARr with the small r standing for “repeat”). The content of the modules is described in Table 2, including a classification of each step into either a more cognitive approach or a more behavioral approach. Based on this, Think and Solve were classified as modules with (primarily) a cognitive approach because the intended outcome of these modules is cognitive change. Act and Relax were classified as modules with (primarily) a behavioral approach because the intended outcome of these modules is behavioral change. In each condition, all four modules were offered, but the sequencing differed per condition (see Design).

**Table 2.** Description of the modules and classification into cognitive and behavioral approaches.

Module	Component	Description of module	Cognitive approach	Behavioral approach
Think	Cognitive restructuring	- Psychoeducation about the relationship between thoughts, feelings, behavior, and short and long-term consequences.	x	
		- Challenging negative thoughts and generating positive thoughts by:		
		(1) identifying negative thought and scheduling; event, thought, feelings, behavior, and consequences;	x	
		(2) assessing credibility of negative thought;	x	
		(3) examining evidence for and against negative thought;	x	
		(4) choosing strongest evidence;	x	
		(5) generating positive thought and scheduling;	x	
		(6) assessing credibility of positive thought;	x	
		(7) reassessing credibility of negative thought.	x	
Solve	Problem solving	- Psychoeducation about problems, coping (avoiding or solving) and consequences. - Solving problems by:	x	
		(1) defining the problem;	x	
		(2) setting a goal;	x	
		(3) generating solutions;	x	
		(4) evaluating solutions (advantages and disadvantages, effectiveness and feasibility);	x	
		(5) choosing a solution and making a plan;	x	x
		(6) conducting the plan;		x
		(7) evaluating the plan and self-reward; - Seeking social support as coping	x	x
Act	Behavioral activation	- Psychoeducation about mood fluctuations, and relationship between mood and activities.	x	x
		- Self-monitoring with daily mood monitors and activity list.		x
		- Examining the link between daily mood and activities in a graph.	x	x
		- Adapting activity list and goal setting to increase pleasant activities.		x
		- Evaluating the goal and new goal setting to increase pleasant activities.	x	x
Relax	Relaxation	- Psychoeducation about stress.	x	x
		- Four types of relaxation exercises: (1) attention/task concentration techniques;	x	x
		(2) breathing techniques;		x
		(3) progressive muscle relaxation;		x
		(4) guided imagery.	x	

With three sessions per module, the total program consisted of 12 sessions of 45–60 min each. Prior to the program, an introductory meeting was provided with psychoeducation about depression and CBT. The STARr-training took place at the participating schools after school lessons, once or twice a week (*M* = 1.27, *SD* = 0.14). The training was provided by 44 pedagogics/psychology graduates (*M*_age_ = 25.84, *SD* = 4.90; 90.9% female; 93.2% Dutch), who were trained and supervised. The mean treatment integrity score for content of the program (e.g., meeting goals) was 84.3% and for form (e.g., time management) was 91.9%. The interrater reliability was substantial (Cohen’s kappa: .69) (see Van den Heuvel et al., 2021).

### Measures

See the protocol paper for all measures that were included in the study (Van den Heuvel et al., 2019). For the current paper, we used the following instruments.

#### Outcome

**Depressive symptoms** were measured with the CDI-2 (Bodden et al., 2016). At baseline, post-intervention, and 6-month follow-up the 28-item version (CDI-2:F) was administered and at intermediate assessment 1 the 12-item version (CDI-2:S). Each item consists of three statements rated in severity of 0 (absent), 1 (sometimes present), or 2 (always present). The CDI-2:F has good psychometric qualities (Bodden et al., 2016). In our study, Cronbach’s alpha at baseline was .86 for the CDI-2:F and .79 for the CDI-2:S. Pearson correlation between the two versions was .95 (*p* < .001).

#### Moderators

**Adolescents’ age (group) and gender** were gathered via self-report at screening. Age was classified into young adolescents (11–14 years; *n* = 185, 65.6%) and older adolescents (15–18 years; *n* = 97, 34.4%). The sample consisted of 157 girls (55.7%) and 125 boys (44.3%).

**Severity of depressive symptoms** was measured with the CDI-2:F (Bodden et al., 2016) at baseline. We classified adolescents into two groups based on the clinical cutoff of 14, namely adolescents with clinical levels of depressive symptoms (raw score ≥ 14; *n* = 162, 57.5%) and adolescents with subclinical levels of depressive symptoms (raw score < 14; *n* = 120, 42.5%).

### Data Analyses

All analyses were conducted in agreement with the intention to treat (ITT) principle, took into account the clustering of participants in treatment groups, and were adjusted for confounders to obtain unbiased estimates. ITT analyses require that missing observations at follow-up were imputed for which we used multiple imputation with chained equations (MICE). Clustering was handled using the first-order Taylor-series linearization method to obtain robust sample errors, *p*-values, and 95% confidence intervals. Despite randomization some baseline imbalances in prognostically important variables may have occurred across conditions. Such confounders were identified and incorporated in the main analyses as covariates.

In order to test whether the effect of the distinct modules and how these have been sequenced collapsed into cognitive and behavioral approaches were moderated by age group, gender, and initial level of depressive symptoms, we conducted regression analyses in Stata (StataCorp, 2019). As a dependent variable we used depressive symptoms (continuous) at intermediate assessment 1 to test the effects of the distinct modules, and depressive symptoms at post-intervention and 6-month follow-up to test the effects of the distinct sequences of modules. As an independent variable, we used the condition variable, which we dichotomized into cognitive approaches (condition 1 and 3) and behavioral approaches (condition 2 and 4). Dummies were created with behavioral approaches as index for the hypotheses related to age group and initial severity level and cognitive approaches as index for the hypotheses related to gender. For all moderators, dummies were created with young adolescents as index for age group, girls as index for gender and adolescents with clinical levels of symptoms as index for severity of depressive symptoms.

To examine the robustness of our findings, all analyses were repeated using the imputation technique estimation maximization (EM).

Contrary to what we planned (see Van den Heuvel et al., 2019), no moderation analyses were conducted for ethnicity, educational level, and level of comorbid problems, because of the very small number of non-Dutch adolescents, the small number of adolescents with a low school level and little variation in comorbid problems in our sample. For all analyses, an alpha level of .05 was used.

## Results

### Age Group

Age group did not moderate the effect on depressive symptoms at intermediate assessment 1 for modules with a behavioral approach versus modules with a cognitive approach, *B* = −0.90, *SE* = 0.55, *p* = .106, 95% CI −2.00 to 0.20. So, young adolescents did not benefit more from the modules Act and Relax than from Think and Solve after three sessions compared to older adolescents.

Also, age group did not moderate the effect on depressive symptoms at post-intervention nor at 6-month follow-up for sequences of modules, starting with a behavioral approach versus sequences starting with a cognitive approach, respectively, *B* = −2.15, *SE* = 1.77, *p* = .237, 95% CI −5.78 to 1.49 and *B* = −3.12, *SE* = 1.88, *p* = .106, 95% CI −6.94 to 0.70. As a result, young adolescents did not benefit more from the sequences *Act*-Think-Relax-Solve and *Relax*-Solve-Act-Think than from *Think*-Act-Relax-Solve and *Solve*-Act-Think-Relax at post-intervention and 6-month follow-up compared to older adolescents.

### Gender

Gender did not moderate the effect on depressive symptoms at intermediate assessment 1 for modules with a cognitive approach versus modules with a behavioral approach, *B* = −0.06, *SE* = 0.59, *p* = .921, 95% CI −1.25 to 1.14. So, girls did not benefit more from the modules Think and Solve than from Act and Relax after three sessions compared to boys.

Also, gender did not moderate the effect on depressive symptoms at post-intervention nor at 6-month follow-up for sequences of modules starting with a cognitive approach versus sequences starting with a behavioral approach, respectively, *B* = −0.81, *SE* = 1.80, *p* = .654, 95% CI −4.47 to 2.84 and *B* = −1.55, *SE* = 2.14, *p* = .474, 95% CI −5.95 to 2.84. As a result, girls did not benefit more from the sequences *Think*-Act-Relax-Solve and *Solve*-Act-Think-Relax than from *Act*-Think-Relax-Solve and *Relax*-Solve-Act-Think at post-intervention and 6-month follow-up compared to boys.

### Initial Severity Level of Depressive Symptoms

Initial severity level did not moderate the effect on depressive symptoms at intermediate assessment 1 for modules with a behavioral approach versus modules with a cognitive approach, *B* = −0.38, *SE* = 0.64, *p* = .560, 95% CI −1.69 to 0.93. So, adolescents with clinical levels of symptoms did not benefit more from the modules Act and Relax than from Think and Solve after three sessions compared to adolescents with subclinical levels of symptoms.

Also, the initial severity level did not moderate the effect on depressive symptoms at post- intervention nor at 6-month follow-up for sequences of modules starting with a behavioral approach versus sequences starting with a cognitive approach, respectively, *B* = 2.58, *SE* = 1.73, *p* = .144, 95% CI −0.93 to 6.08 and *B* = 1.59, *SE* = 1.82, *p* = .389, 95% CI −2.10 to 5.28. As a result, adolescents with clinical levels of depressive symptoms did not benefit more from the sequences *Act*-Think-Relax-Solve and *Relax*-Solve-Act-Think than from *Think*-Act-Relax-Solve and *Solve*-Act-Think-Relax at post-intervention and 6-month follow-up compared to adolescents with subclinical levels of symptoms.

### Sensitivity Analyses

Sensitivity analyses revealed comparable results (no significant moderator effects) attesting to the robustness of the findings.

## Discussion

With the current study, we aimed to provide insight into subgroups of adolescents that benefit more or to a lesser extent from predominantly cognitive versus predominantly behavioral approaches to preventing depression. Age group, gender, and initial severity of depressive symptoms were investigated as moderators. The ultimate goal is to optimize CBT in indicated depression prevention for specific subgroups of adolescents.

Contrary to our hypotheses, we found that age group, gender, and initial severity level did not moderate the effects of modules based on a more cognitive approach (Think and Solve) versus modules based on a more behavioral approach (Act and Relax) on depressive symptoms after three sessions (relative to baseline). A previous study showed that none of the distinct modules were associated with a significant reduction in depressive symptoms after three sessions (Van den Heuvel et al., 2021). The current study showed that there were no differences in (the absence of) effect of specific modules when provided to younger or older adolescents, girls or boys, and adolescents with clinical or subclinical levels of depressive symptoms. Therefore, regardless of a more cognitive or a more behavioral approach, a single module of three sessions is not sufficient to reduce depressive symptoms among all subgroups of adolescents that were investigated in this paper.

Age group, gender, and initial severity level did also not moderate the effects of sequences starting with a cognitive approach (*Think*-Act-Relax-Solve and *Solve*-Act-Think-Relax) versus sequences starting with a behavioral approach (*Act*-Think-Relax-Solve and *Relax*-Solve-Act-Think) on depressive symptoms at post-intervention and 6-month follow-up (relative to baseline), which also contradicts our hypotheses. Previously, we found that all sequences of modules were significantly associated with a reduction in depressive symptoms at post-intervention (except for the sequence Relax-Solve-Act-Think, CDI-2 full version) and 6-month follow-up (Van den Heuvel et al., 2021). The current study showed that these effects did not differ between younger or older adolescents, girls or boys, and adolescents with clinical or subclinical levels of depressive symptoms. Therefore, regardless of the sequencing of CBT components (a more cognitive based versus a more behavioral based start), the four components together reduced depressive symptoms among all subgroups of adolescents that were investigated in this paper.

In summary, we find no evidence for substantial moderation effects (approximately *d* ≥ 0.46) and will cautiously assume that the intervention is not moderated by the variables studied. Therefore, regardless of age group, gender, and initial severity level, a single module of three sessions (regardless of their CBT component) is not sufficient to reduce depressive symptoms, while the four modules together (regardless of their sequence) are sufficient in reducing depressive symptoms in indicated prevention among adolescents. This is in line with the literature examining moderating effects in “whole CBT packages” which show that, in general, age and gender do not moderate response to CBT in depression prevention among adolescents compared to diverse control groups (e.g., Conejo-Cerón et al., 2020). The moderating role of the initial severity level of depressive symptoms of “whole CBT packages” in depression prevention among adolescents is unclear, with some studies showing no moderator effect and other studies showing contradictory results (e.g., Conejo-Cerón et al., 2020). Our study indicated no moderating role of severity level in the effect of the distinct cognitive versus behavioral modules and the more cognitive versus behavioral start of different sequences. However, it is important to note that all participants were required to have elevated levels of depressive symptoms. This might have restricted the range of depressive symptom severity, which might have contributed to the non-significant effects for this moderator in our study.

The aim of moderation analyses is to examine whether or not the effects of an intervention are comparable across different subgroups of adolescents. The results of the current study suggest that the effects of more cognitive based versus more behavioral based CBT-modules after three sessions and the effect of a cognitive versus a behavioral based start of different sequences at post-intervention and 6-month follow-up apply to a relatively wide range of adolescents in terms of age group, gender, and severity level of symptoms (in indicated prevention). This provides opportunities for more modular and personalized interventions, wherein the content of the program (sequencing of modules) can be adapted to the individual or the group, based on what is deemed most relevant and/or preferable according to the client and trainer. One example of such a modular approach is Modular Approach to Therapy for Children with Anxiety, Depression, or Conduct Problems (MATCH) (Chorpita & Weisz, 2009), which has shown promising results (summarized in Van den Heuvel et al., 2021).

To our knowledge, this is the first study that aimed to identify moderators in the effect of individual CBT components and different sequences in adolescent depression prevention. Although we found no significant moderator effects, null-findings are important to report as null-findings among moderators could mean that the intervention can be widely used. Another strength of this study is that the effects were examined over three timepoints, namely after the first module, at post-intervention and 6-month follow-up.

Our study also has limitations. First, the null-findings of our study should be interpreted with caution, as absence of evidence for moderation should not be confused with evidence for the absence of moderation. With the current sample size, we were only able to detect moderator effects of medium size (*d* ≥ 0.46), thus obscuring smaller effects, if any. A second limitation is that we used a “one at a time” analytic approach to moderation, which have recently been criticized for their likely limited explanatory power regarding response to treatment (Mullarkey & Schleider, 2021). It is more likely that many factors determine an individual’s response to treatment. Third, since our sample was relatively homogeneous in terms of ethnicity (mostly Dutch), school level (mostly higher levels), and comorbid problems (few comorbid problems), these variables could not be included in the moderation analyses although this was originally planned. Fourth, the separate modules consisted of only three sessions, which might not have been sufficient to change depressive symptoms and reveal any subgroup differences. Dose–response studies could reveal the optimal dose of the different modules in general and in relationship to different subgroups. For example, it could be that some subgroups of adolescents need more sessions of a specific module than other subgroups. Finally, the analyses focused on identifying differential responses to two types of treatment sequences. Although the sequences of modules differed, the content was similar (each sequence consisted of the same modules), so they may not have been different enough to demonstrate moderation effects.

Additional studies with more power are required to replicate these findings. Besides, other possible moderators in relation to distinct CBT components and sequences (whether or not offered flexibly) should be investigated, such as other clinical variables (e.g., comorbid problems, emotion regulation strategies, level of cognitive errors, level of behavioral activation, stress level, and problem solving skills) and/or interpersonal functioning (e.g., social support), all in order to provide more knowledge on what works best for whom. We also recommend to use more person-centered methodological approaches, which can identify subgroups of adolescents based on their similarities on multiple variables, as this is highly relevant for clinical practice (Bergman & Magnusson, 1997; Mullarkey & Schleider, 2021). One way to do this is by generating latent profiles based on multiple variables (e.g., by using latent profile analyses, see for example Van den Heuvel et al., 2020) and to examine whether these profiles moderate the effect of the intervention (components). Another way is to use more advanced designs, such as Sequential Multiple Assignment Randomized Trial (SMART). SMART is a design in which a client is initially randomized to a condition (e.g., a CBT module) and is re-randomized at a subsequent stage based on interim results (e.g., responding or non-responding) (Collins et al., 2007). This design is relevant in the question of heterogeneity in treatment response because it takes into account possible individual differences from the start until the end and provides insight into what works for whom.

Several clinical implications may be derived from our findings. The investigated indicated prevention program STARr might be used for a broad range of adolescents with elevated depressive symptoms in Dutch secondary schools, making implementation and dissemination easier. Since an increase in the prevalence of depression is expected as a consequence of the COVID-19 crisis (Green et al., 2021; Holmes et al., 2020), short personalized and accessible interventions are important. The four STARr modules might be used flexibly regarding their sequencing, without decreasing effects, among adolescents of different age, gender, and level of depressive symptoms. Choices regarding the sequencing can be made strategic, for example based on the main problems of the adolescents. But it also gives room for the clinician to start with the module in which the clinician is most competent in order to make the biggest impact in achieving treatment effect early in the intervention.

## Conclusion

This study tested the moderating role of age group, gender, and severity level of depressive symptoms in the effects of CBT-modules based on a more cognitive approach versus a more behavioral approach after three sessions and the effects of sequences starting with a more cognitive approach versus a more behavioral approach at post-intervention and 6-month follow-up in indicated depression prevention among Dutch adolescents. Overall, results showed that none of these variables moderated the effects of cognitive versus behavioral based modules after three sessions and cognitive versus behavioral based sequences at post-intervention and 6-month follow-up. These findings provide opportunities for more modular approaches in adolescent depression prevention wherein the content can be personalized based on the adolescents’ and/or trainers’ preferences.

## Trial Registration

Dutch Trial Register (NL5584/NTR6176)
